# Identification and Validation of Reference Genes for Quantitative Real-Time PCR in *Drosophila suzukii* (Diptera: Drosophilidae)

**DOI:** 10.1371/journal.pone.0106800

**Published:** 2014-09-08

**Authors:** Yifan Zhai, Qingcai Lin, Xianhong Zhou, Xiaoyan Zhang, Tingli Liu, Yi Yu

**Affiliations:** 1 Institute of Plant Protection, Shandong Academy of Agricultural Sciences, Jinan, China; 2 College of Plant Protection, Shandong Agricultural University, Taian, China; 3 College of Plant Protection, Yunnan Agricultural University, Kunming, China; Institute of Vegetables and Flowers, Chinese Academy of Agricultural Science, China

## Abstract

To accurately evaluate gene expression levels and obtain more accurate quantitative real-time RT-PCR (qRT-PCR) data, normalization relative to reliable reference gene(s) is required. *Drosophila suzukii*, is an invasive fruit pest native to East Asia, and recently invaded Europe and North America, the stability of its reference genes have not been previously investigated. In this study, ten candidate reference genes (*RPL18*, *RPS3*, *AK*, *EF-1β*, *TBP*, *NADH*, *HSP22*, *GAPDH*, *Actin*, *α-Tubulin*), were evaluated for their suitability as normalization genes under different biotic (developmental stage, tissue and population), and abiotic (photoperiod, temperature) conditions. The three statistical approaches (geNorm, NormFinder and BestKeeper) and one web-based comprehensive tool (RefFinder) were used to normalize analysis of the ten candidate reference genes identified *α-Tubulin*, *TBP* and *AK* as the most stable candidates, while *HSP22* and *Actin* showed the lowest expression stability. We used three most stable genes (*α-Tubulin*, *TBP* and *AK*) and one unstably expressed gene to analyze the expression of P-glycoprotein in abamectin-resistant and sensitive strains, and the results were similar to reference genes *α-Tubulin*, *TBP* and AK, which show good stability, while the result of *HSP22* has a certain bias. The three validated reference genes can be widely used for quantification of target gene expression with qRT-PCR technology in *D.suzukii*.

## Introduction

Quantitative real-time PCR (qRT-PCR) is a fast, sensitive, repeatable and accurate method for quantifying gene transcript levels [1], [2]. However, several studies have revealed that interpretation would produce appreciable errors owing to different samples, treatments, RNA extraction techniques, amplification efficiencies, etc. Therefore, it requires an appropriate candidate reference gene to normalize the target gene expression, and the reference genes must have stable expression existing features in different cell types and experimental conditions [3]–[5].

Spotted wing drosophila (SWD), *Drosophila suzukii* (Diptera: Drosophilidae), is one of a handful of species that live on undamaged ripening fruits, and using its peculiar serrated ovipositor to break the skin of fresh ripening fruits and lay eggs in it [6]. *D. suzukii* was first recorded in Eastern Asia, and its western invasion started in 2008 with synchronous outbreaks in North America and Europe, which seriously threatened the local fruit industry, and has inflicted huge financial losses [7]. At present, there are few research reports on *D. suzukii*, and so far no qRT-PCR studies on it have been reported, so there is especially important to validated reference gene(s) for it.

So far, several housekeeping genes, such as ribosomal protein L18 (*RPL18*) [8], ribosomal protein S3 (*RPS3*) [9], arginine kinase (*AK*) [10], elongation factor 1 beta (*EF-1β*) [11], TATA binding protein (*TBP*) [12], NADH dehydrogenase (*NADH*), heat shock protein 22 (*HSP22*) [13], glyceraldehyde-3-phosphate dehydrogenase (*GAPDH*) [14], *Actin* [15] and *α-Tubulin* [16], [17] have been used widely as reference genes for qRT-PCR analysis in Insecta and other species. However, based on previous studies, it will not have a single universal reference gene that is suited for all experimental conditions [18]–[20]. Therefore, it is especially important to select the reliable reference genes in qRT-PCR based transcriptome studies.

In order to select the most suitable reference gene(s) for gene expression quantification by qRT-PCR, we examined the expressions of ten candidate reference genes (*RPL18*, *RPS3*, *AK*, *EF-1β*, *TBP*, *NADH*, *HSP22*, *GAPDH*, *Actin*, *α-Tubulin*) in four developmental stages (egg, larvae, pupa, and adult), three body regions (head, thorax, and abdomen), three different populations, and two abiotic conditions (photoperiod, temperature) treated samples. The gene expression quantification data were assessed using three statistical approaches (geNorm, NormFinder and BestKeeper) to normalized analysis.

## Materials and Methods

### Insects

The laboratory strain of *D. suzukii* was collected in cherry fields at Tai'an in Shandong Province, China in 2012. The solution of sucrose was serving as a food and water source for adults, and larvae were reared on a banana medium, which also served as ovipositional substrate. The rearing process was maintained in a greenhouse at a temperature of 25±1°C, 16: 8 h light: dark photoperiod and 70–80% relative humidity. The abamectin-resistant strain was derived from the laboratory (abamectin-sensitive) strain, which feed on medium containing progressively higher doses of abamectin for more than 18 months. The species are common agricultural pests and not included in the “List of Protected Animals in China”. No specific permissions were required as these fields are experimental plots that belong to Shandong Academy of Agricultural Sciences, Jinan, Shandong in China.

### Reference Gene Selection and Primer Design

Ten commonly used reference genes were selected, based on the described insect reference genes in literature, the Spotted Wing Fly Base (http://spottedwingflybase.oregonstate.edu) was searched for available *D. suzukii* sequences: *RPL18*, *RPS3*, *AK*, *EF-1β*, *TBP*, *NADH*, *HSP22*, *GAPDH*, *Actin*, *α-Tubulin*. Primer 5.0 (http://www.premierbiosoft.com/) was used to design primers for qRT-PCR analysis. The gene characteristics and primer sequences were summarized in Table 1.

**Table 1:** Primers used for qRT-PCR analysis.

Gene	Gene ID^a^	Primer sequences (5′to 3′)^b^	Product length (bp)	Tm (°C)	Primer efficiency (%)	R^2c^
**ribosomal protein L18**	DS10_00006641	F: GTTGCTCCAAACCCTCCA	117	60	92.6	0.9988
***RPL18***		R: GATCCGTCTAACACCTCCC				
**ribosomal protein S3**	DS10_00012475	R: GTGCTCGGCATCAAGGTC	100	60	108.0	0.9978
***RPS3***		F: TGGGCTCCACAACAGACAC				
**arginine kinase**	DS10_00003811	F: CTACCACAACGATGCCAAGA	180	60	109.4	0.9994
***AK***		R: AAGGTCAGGAAGCCGAGA				
**elongation factor 1 beta**	DS10_00003263	F: AAGAAGCCCGCCCTCATC	123	60	98.5	0.9909
***EF-1β***		R: CCACAGCAGACCGTCCATC				
**TATA binding protein**	DS10_00003466	F:CCACGGTGAATCTGTGCT	182	60	101.3	0.9987
***TBP***		R:GGAGTCGTCCTCGCTCTT				
**NADH dehydrogenase**	DS10_00011622	F: CGAGGTTGTGGTGGAGGA	227	60	99.5	0.9945
***NADH***		R: CATCACGCCAGACTTTGCT				
**heat shock protein 22**	DS10_00003839	F: CTGGTGGAGGGCAAATCG	127	60	95.0	0.9973
***HSP22***		R: CGCTGCTCAGACTGGAGGT				
**glyceraldehyde-3-phosphate dehydrogenase**	DS10_00002887	F: GGTCCTTCGGGCAAACTG	217	60	105.6	1.0000
***GAPDH***		R: CCTTAGCCTTGATCTCATCGTA				
***Actin***	DS10_00010769	F:TCTTCCAGCCCTCGTTCC	109	60	107.3	0.9899
		R:TTGTTGGCATACAGGTCCTTAC				
***α-Tubulin***	DS10_00003884	F: AGGATGCGGCGAATAACT	189	60	96.7	0.9963
		R: CGGTGGATAGTCGCTCAA				

a the Spotted Wing Fly Base (http://spottedwingflybase.oregonstate.edu);

b F and R refer to forward and reverse primers, respectively;

c R^2^ refers to the coefficient of determination.

### Biotic Factors

#### Developmental stages

Four developmental stages (egg, larvae, pupa, and adult) were collected from *D. suzukii.* Samples used comprised 300 eggs, 30 first-instar larvae, 30 second-instar larvae, 20 third-instar larvae, 20 pupae, 20 first-day male and female adults, 20 seven-day old male and female adults and 20 fifteen-day old male and female adults for each replication. All the samples were immediately frozen in liquid nitrogen and stored at −80°C.

#### Tissue

Three body regions (head, thorax, and abdomen) were obtained from seven-day old adults using dissection needle and a stereo microscope in PBS solution on ice.

#### Population

One laboratory *D. suzukii* strain and two field collected populations from Zhejiang and Yunnan provinces were used.

### Abiotic Conditions

#### Photoperiod

Each group of 10 seven-day old adults was kept in glass chambers with photoperiods (L/D) of 8∶16, 12∶12, and 16∶8 for 72 h, and then the live adults stored for RNA extraction.

#### Temperature

A total of 30 seven -day-old adults were collected and placed individually into glass tubes. The tubes were then placed in water bath at 15.0, 25.0, and 35.0°C for 1 h, and stored as described earlier.

### Total RNA Isolation and cDNA Synthesis

Total RNA was isolated with Total RNA Kit II (Omega, USA), RNA quantity was evaluated using a microvolume spectrophotometer NanoDrop 2000 (Thermo, USA), and the integrity was checked with 1% agarose gel electrophoresis. First-strand cDNA was synthesized with the PrimeScript RT reagent Kit (TaKaRa, Japan), the synthesized cDNAs were stored at −20°C for further qRT-PCR.

### Quantitative Real-time PCR

The synthesized cDNA was amplified by PCR in 10 µL reaction mixtures using a Light Cycler 480 system (Roche, USA) and SYBR Premix Ex Taq (Takara, Japan) with the following procedure: 94°C for 5 min, followed by 45 cycles of 94°C for 15 s, 60°C for 20 s, and 72°C for 15 s. After the amplifications, a melting curve analysis was performed in triplicate, and the results were averaged. A 10-fold dilution series of cDNA was used to create the standard curve, and the qRT-PCR efficiency was determined for each gene and each treatment used to convert the Ct-values into the relative quantities.

### Reference Gene Validation

P-glycoprotein, an ATP-dependent drug-efflux pump transmembrane protein, which closely related to abamectin resistance in *Drosophila* [21]. P-glycoprotein (DS10_00005769) was selected as the target gene to validate the reference genes. (P-glycoprotein F: AGCGTCACGACAAGAGGA; R: ACCACCCACAACGAGGAA). The relative expression level of the target gene was calculated with different normalization factors, the selected most/least stable genes, and which was determined in abamectin-resistant and sensitive strains.

### Statistical Analysis

Three software programs, geNorm (version 3.4), NormFinder (version 0.953) and BestKeeper (version 1), were used to evaluate the stability of the ten candidate reference genes. The geNorm program calculates an expression stability value (M) for each gene, the lower the M values, and the more stable reference genes. And then compares the pair-wise variation (V) of this gene with the others, we used pairwise variation V_n/n+1_ to estimate the optimal number of reference genes. A value below 0.15 indicates that an additional reference gene will not significantly improve normalization [14]. NormFinder uses a model-based approach to evaluate the overall variation of the candidate reference genes, the lower values, the more stable reference genes [22]. BestKeeper uses the geometric mean of the Ct values of the candidate reference genes with a standard deviation (SD), the lower index scores, and the more stable reference genes [23].

RefFinder (http://www.leonxie.com/referencegene.php?type=reference) is a web-based comprehensive tool that was used to evaluate optimal reference genes by integrating the results of the previous three analyses. Finally, we selected the ideal reference genes determined by RefFinder to choose the most stable genes [24].

## Results

### Total RNA Quality and PCR Amplification Efficiencies

The concentration and purity of RNA isolated from different samples were determined using the microvolume spectrophotometer NanoDrop 2000, the absorbance ratios at 260/280 above 1.80 for all RNA samples. The integrity of all total RNA samples was confirmed using 1.0% agarose gel electrophoresis.

For each reference gene, a melting curve with a single peak suggested that each of the primer pairs amplified a unique product ranging from 100 (*RPS3*) to 227 bp (*NADH*; Fig. S1). The primer efficiencies of each standard curve for PCR efficiency, which was generated from third-instar larvae of the laboratory strain, were ranging from the lowest for *RPL18* (92.6%) to the highest for *AK* (109.4%). The linear regression coefficients were larger than 0.99 for candidate reference genes except *Actin* (0.9899; Table 1). Altogether, these results confirmed that the selected primers accurately amplify candidate reference genes.

### Expression Profiles of Reference Genes

**Figure 1:**
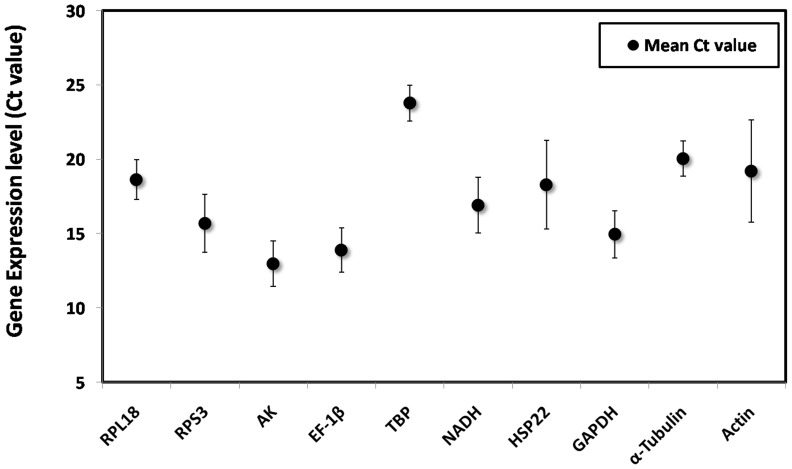
Expression levels of candidate reference genes in different samples of *D.suzukii*. Expression levels are displayed as cycle threshold (Ct) values of the candidate reference genes used in this study. Data represent mean values ± S.E.M. (n = 150).

The cycle threshold values analysis of candidate reference genes in all different samples ranged from 11.01 to 27.21. Of the tested genes, the Ct values ranged from 12.32 (*AK*) to 26.25 (*TBP*) for different developmental stages, from 11.01 (*AK*) to 23.46 (*TBP*) for different tissues, from 14.63 (*AK*) to 27.21 (*TBP*) for different populations, from 13.35 (*AK*) to 21.73 (*TBP*) with different photoperiods, from 13.57 (*AK*) to 20.25 (*TBP*) with different temperatures. The Ct values showed that *AK* was the most abundant transcript with a Ct value of 12 or lower, whereas *TBP* was the least abundantly transcribed. Based on Ct dispersion, all candidate genes except *HSP22* and *Actin* exhibited relatively small variations (Fig. 1).

### Stability of Reference Gene Expression

#### geNorm Analysis

**Figure 2:**
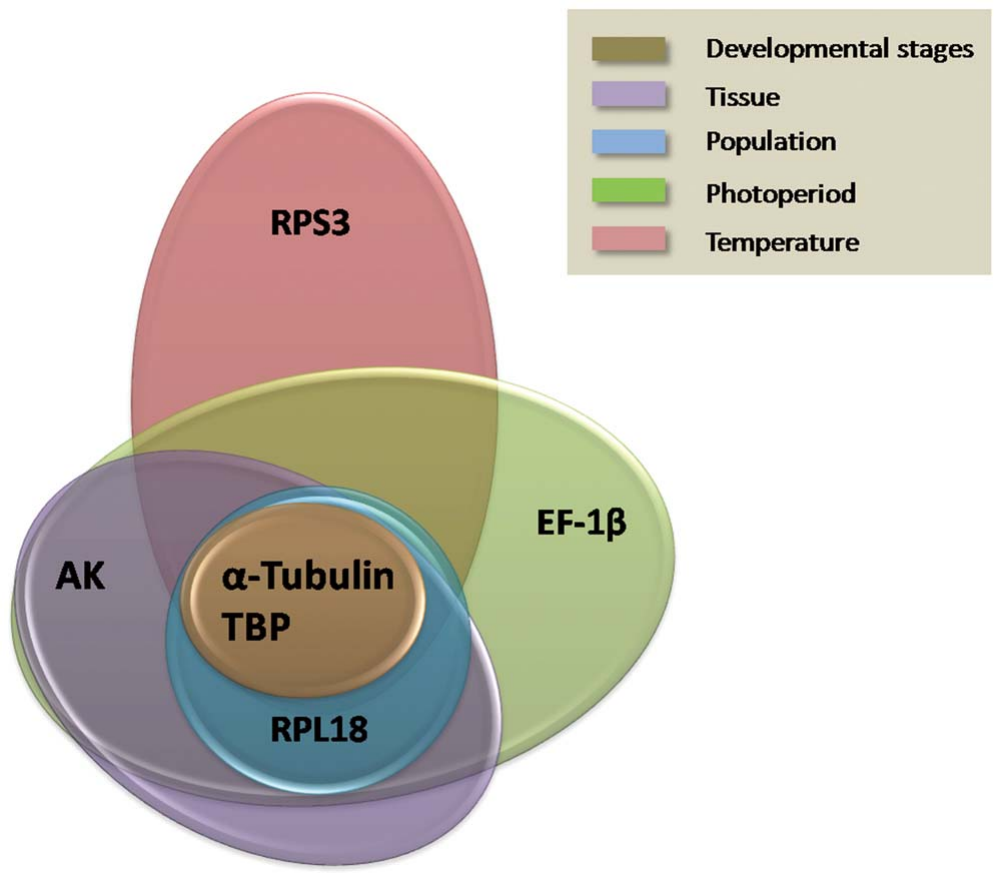
Venn diagrams showing the most stable genes identified by geNorm. The most stable genes were identified using data from various biotic and abiotic conditions. Each circle with a distinct color represents a different condition.

The geNorm program was used to calculate the average expression stability values (M values). The M values of tested genes was lower, the stability expression was higher, and vice versa. Considering the data obtained from various biotic conditions. For developmental stages, *α-Tubulin* and *TBP* were found to be the two most stable genes with the lowest M values. Similarly, *HSP22* was found to be the least stable gene with the highest M value (Fig. S2A). For tissue, the *AK* and *RPL18* genes showed the greatest expression stability, while *Actin* showed the least expression stability (Fig. S2B). For population, *α-Tubulin* and *TBP* were expressed more stable than other candidate reference genes, while *Actin* was expressed less stably than all others (Fig. S2C). Under various abiotic conditions, for photoperiod, *α-Tubulin* and *RPL18* were the most stably expressed genes, while *HSP22* was the least stably expressed gene (Fig. S2D). For temperature, the ranking of reference gene stability among those with M values <0.5 is *RPS3*, *TBP* and *α-Tubulin* (Fig. S2E). Based on data obtained with five biotic and abiotic factors, *α-Tubulin* and *TBP* may be suitable for gene expression analysis in this study (Fig. 2).

**Figure 3:**
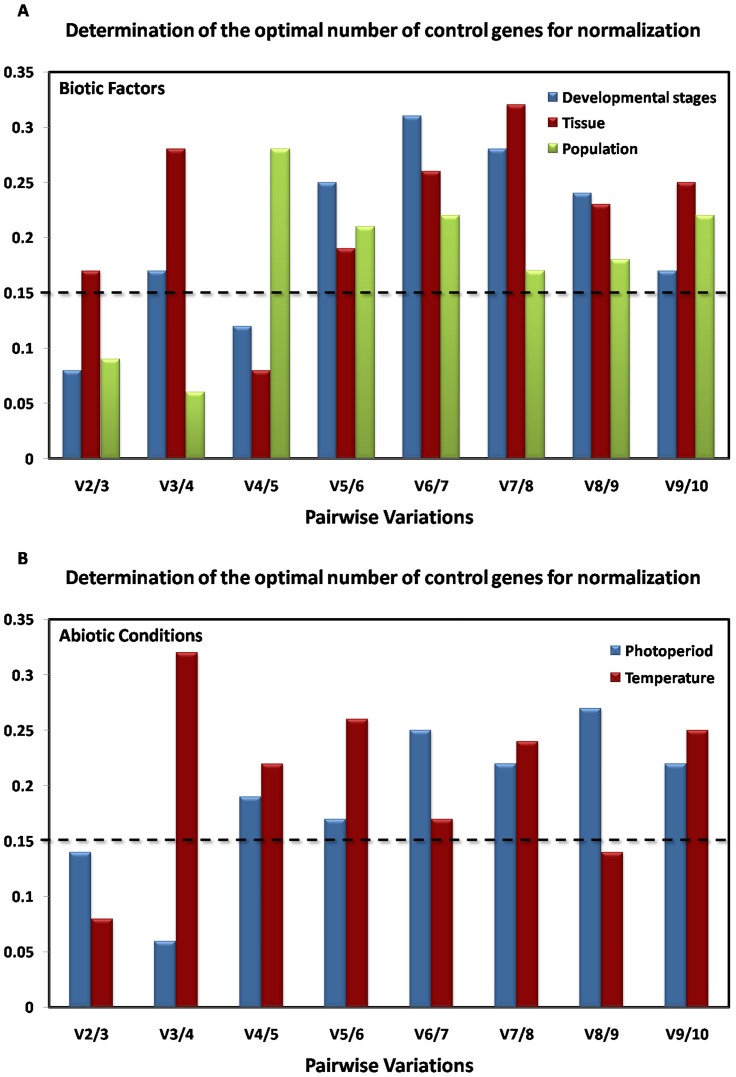
The optimal number of reference genes for normalization by geNorm analysis. Optimal number of reference genes required for accurate normalization of gene expression under biotic (A) and abiotic (B) conditions. Average pairwise variations (V) were calculated between the normalization factors NF_n_ and NF_n+1_ to indicate whether inclusion of an extra reference gene adds to the stability of the normalization factor.

In addition, geNorm was used to calculate the optimal number of control genes for normalization. If the pairwise variation of Vn/n+1<0.15, it is not necessary to use ≥ n+1 genes as internal controls. Accordingly, the pairwise variation of V2/3 was lower than 0.15 in most of the conditions, except different tissues (Fig. 3). This indicates that combined use of the two stable control genes would be suitable for this research.

#### NormFinder Analysis

As with the geNorm method, the gene with the lowest M value has the most stable expression, and the NormFinder analysis result was similar to the result of geNorm analysis. NormFinder indicated that *α-Tubulin*, *TBP* and *RPL18* are the most stable reference genes, and *Actin* and *HSP22* are the least stable expression for five biotic and abiotic conditions (Table 2).

**Table 2:** Ranking of candidate reference genes in order of their expression stability as calculated by NormFinder.

Rank	Biotic Conditions						Abiotic Conditions			
	Developmental stages		Tissue		Population		Photoperiod		Temperature	
	Gene	SV^a^	Gene	SV	Gene	SV	Gene	SV	Gene	SV
**1**	*α-Tubulin*	0.513	*RPL18*	0.173	*TBP*	0.352	*RPL18*	0.382	*TBP*	0.480
**2**	*TBP*	0.637	*AK*	0.302	*Α-Tubulin*	0.441	*α-Tubulin*	0.432	*RPS3*	0.502
**3**	*RPL18*	0.692	*α-Tubulin*	0.379	*AK*	0.560	*TBP*	0.475	*α-Tubulin*	0.681
**4**	*AK*	0.746	*NADH*	0.481	*NADH*	0.598	*RPS3*	0.552	*RPL18*	0.762
**5**	*GAPDH*	0.903	*TBP*	0.523	*RPL18*	0.731	*GAPDH*	0.735	*NADH*	0.807
**6**	*RPS3*	0.958	*EF-1β*	0.720	*EF-1β*	0.953	*AK*	0.856	*GAPDH*	0.890
**7**	*EF-1β*	1.030	*RPS3*	0.815	*GAPDH*	1.136	*NADH*	0.993	*AK*	0.953
**8**	*NADH*	1.433	*HSP22*	1.169	*RPS3*	1.169	*EF-1β*	1.259	*Actin*	1.105
**9**	*Actin*	1.495	*GAPDH*	1.383	*HSP22*	1.401	*HSP22*	1.540	*EF-1β*	1.118
**10**	*HSP22*	1.732	*Actin*	1.425	*Actin*	1.802	*Actin*	1.763	*HSP22*	1.368

a SV refers to the stability value.

#### BestKeeper Analysis

The BestKeeper program is another software tool to calculate the stability of a candidate reference gene, based on the standard deviation (SD) of the Ct values. The most stable reference genes were identified based on having the lowest standard deviation (SD). In this study, the BestKeeper analyses indicated that *TBP* and *α-Tubulin* were the most stably expressed genes, and *HSP22* was the least stably expressed genes for different developmental stages (Table 3). In different tissues samples, the most stable genes were *Actin* and *α-Tubulin*, while *HSP22* had the highest SD of all of the selected genes. Among the different populations, the most stable reference genes were *TBP and TBPα-Tubulin*, with the lowest SD values. In the different photoperiod-treated models, the most stable genes were found to be *TBP* and *AK*, and the least stable gene was found to be *HSP22*. For temperature, *TBP* and *NADH* were expressed more stable than other candidate reference genes.

**Table 3:** Ranking of candidate reference genes in order of their expression stability as calculated by BestKeeper.

Rank	Biotic Conditions						Abiotic Conditions			
	Developmental stages		Tissue		Population		Photoperiod		Temperature	
	Gene	SD^a^	Gene	SD	Gene	SD	Gene	SD	Gene	SD
**1**	*TBP*	0.30	*Actin*	0.34	*TBP*	0.20	*TBP*	0.08	*TBP*	0.16
**2**	*α-Tubulin*	0.44	*Α-Tubulin*	0.36	*α-Tubulin*	0.34	*AK*	0.12	*NADH*	0.36
**3**	*AK*	0.61	*NADH*	0.37	*NADH*	0.34	*α-Tubulin*	0.27	*α-Tubulin*	0.40
**4**	*RPL18*	0.72	*AK*	0.39	*RPL18*	0.42	*NADH*	0.36	*AK*	0.49
**5**	*GAPDH*	0.88	*RPL18*	0.45	*EF-1β*	0.54	*EF-1β*	0.43	*RPL18*	0.56
**6**	*NADH*	0.96	*TBP*	0.58	*AK*	0.68	*RPL18*	0.44	*RPS3*	0.62
**7**	*EF-1β*	1.16	*EF-1β*	0.65	*GAPDH*	1.23	*RPS3*	0.91	*HSP22*	0.65
**8**	*RPS3*	1.32	*RPS3*	0.70	*RPS3*	1.30	*Actin*	1.24	*EF-1β*	0.69
**9**	*Actin*	1.37	*GAPDH*	0.96	*HSP22*	1.84	*GAPDH*	1.43	*GAPDH*	1.00
**10**	*HSP22*	1.50	*HSP22*	1.02	*Actin*	2.35	*HSP22*	2.09	*Actin*	1.17

a SD refers to the standard deviation.

#### RefFinder Analysis

Based on the geometric mean (GM) of the rankings obtained from three complementary statistical approaches, the five most reliable reference genes were the same for biotic or abiotic conditions samples. However, their ranks were a little different between biotic and abiotic samples. *α-Tubulin* (GM = 1.32)was the preferred candidate in developmental stages. The most stable to the least stable in the different tissues samples was *TBP*, *AK*, *α-Tubulin*, *NADH* and *EF-1β*. Among the different populations, *α-Tubulin* (GM = 1.41) was the most stably expressed gene, the remaining ranked candidates were *TBP*, *EF-1β*, *NADH* and *AK* with GM values ranging from 2.54 to 4.66, respectively. For photoperiod, *α-Tubulin* (GM = 1.41) and *TBP* (GM = 2.52) were expressed more stable than other candidate reference genes. For temperature, the order of best reference genes was as follows: *TBP* (GM = 1.00), *AK* (GM = 1.54), *α-Tubulin* (GM = 2.42), *EF-1β* (GM = 2.61), and *NADH* (GM = 3.54) (Table 4).

**Table 4:** Ranking of candidate reference genes in order of their expression stability as calculated by RefFinder.

Rank	Biotic Conditions						Abiotic Conditions			
	Developmental stages		Tissue		Population		Photoperiod		Temperature	
	Gene	GM^a^	Gene	GM	Gene	GM	Gene	GM	Gene	GM
**1**	*α-Tubulin*	1.32	*TBP*	1.00	*Α-Tubulin*	1.41	*α-Tubulin*	1.41	*TBP*	1.00
**2**	*TBP*	2.11	*AK*	2.35	*TBP*	2.54	*TBP*	2.52	*AK*	1.54
**3**	*AK*	2.54	*α-Tubulin*	3.45	*EF-1β*	2.85	*AK*	3.24	*α-Tubulin*	2.42
**4**	*EF-1β*	3.51	*NADH*	4.21	*NADH*	3.52	*NADH*	3.86	*EF-1β*	2.61
**5**	*NADH*	4.00	*EF-1β*	4.95	*AK*	4.66	*EF-1β*	4.52	*NADH*	3.54
**6**	*RPS3*	5.23	*RPS3*	5.35	*RPL18*	5.48	*RPS3*	6.42	*Actin*	4.63
**7**	*RPL18*	5.71	*RPL18*	6.62	*RPS3*	6.82	*GAPDH*	7.51	*RPS3*	6.82
**8**	*GAPDH*	6.72	*HSP22*	7.75	*Actin*	7.56	*RPL18*	8.23	*RPL18*	7.56
**9**	*Actin*	8.15	*GAPDH*	8.54	*GAPDH*	8.64	*HSP22*	8.85	*GAPDH*	8.35
**10**	*HSP22*	9.54	*Actin*	9.15	*HSP22*	8.84	*Actin*	9.68	*HSP22*	10.23

a GM refers to the Geometric mean.

### Reference Gene Validation

**Figure 4:**
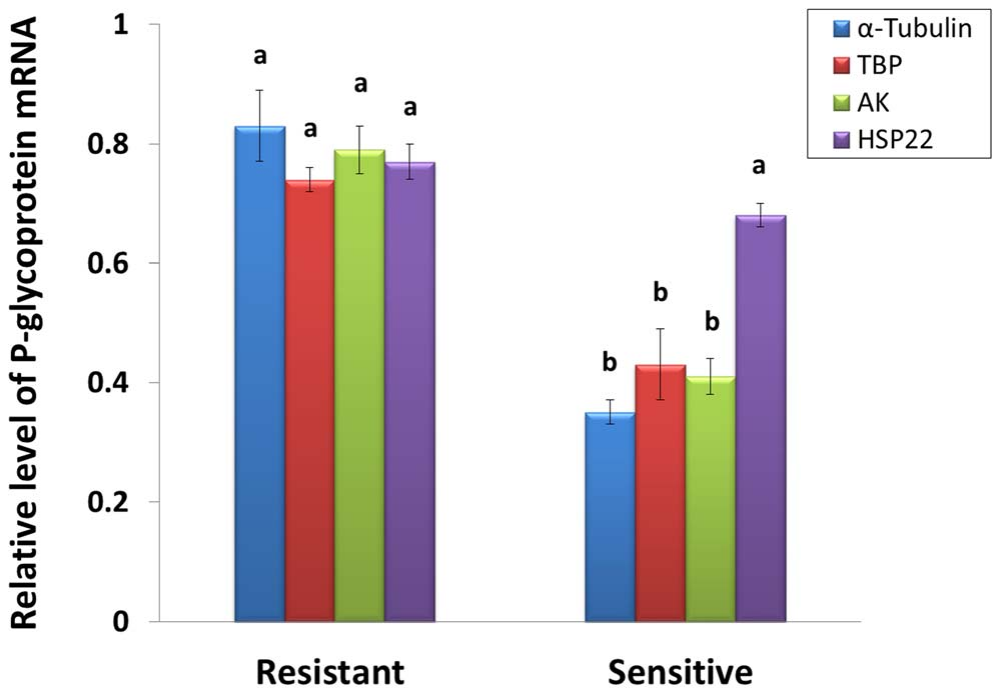
The expression levels of P-glycoprotein by qRT-PCR. The transcript levels of P-glycoprotein gene in abamectin-resistant and sensitive strains by qRT-PCR using four different reference genes. Data represent mean values ± S.E.M, and those in the columns followed by the different letters mean significant difference (*p* = 0.05, Duncan's multiple range test).

P-glycoprotein expression levels was higher in resistant than in susceptible strain had been previously found by Lou *et al* [21] using Western blotting and Vanadate-sensitive ATPase assay. P-glycoprotein gene was further assessed by using the reference genes *α-Tubulin*, *TBP* and *AK* in comparison to *HSP22*. The expression profiles of the P-glycoprotein gene obtained using these three stable reference genes showed a similar trend in resistant or susceptible strain, but when *HSP22* was used as the reference for normalization, the expression profile of P-glycoprotein gene changed. At the same time, there was significant expression difference of P-glycoprotein, which was higher in resistant than in susceptible strain, when calculated with *α-Tubulin*, *TBP* and *AK* as internal control, further demonstrating that *α-Tubulin*, *TBP* and *AK* were adequate internal controls On the contrary, there was no significant difference of the target gene, when used the least stable gene *HSP22* (Fig. 4). This indicated the questionable results would be to produce by using unstable reference gene.

## Discussion

Due to its high sensitivity, accuracy, specificity and repeatability, qRT-PCR is the first choice tool for gene expression analysis. Gene expression can vary across different physiological stages or cell types, so an appropriate internal control gene is required. The ideal reference gene should have relatively stable expression in distinct samples and different experimental conditions. In this study, we used three algorithms (geNorm, NormFinder and BestKeeper) and one web-based comprehensive tool (RefFinder) to evaluate the stability of 10 candidate reference genes in *D.suzukii* in response to different biotic (developmental stage, tissue and population), and abiotic (photoperiod, temperature) conditions.

Considering the developmental stages, the most stable genes were *α-Tubulin* and *TBP* (geNorm, NormFinder and BestKeeper). For different tissues, *AK* and *RPL18* were identified as the two most stable genes by geNorm and NormFinder, while the two most stable genes by BestKeeper analysis were *α-Tubulin* and *Actin*. Among different populations, *TBP* and *α-Tubulin* had constant expression in all data sets produced by the three algorithms. In the different photoperiod-treated models, *α-Tubulin* and *RPL18* were identified more stable genes by geNorm and NormFinder, while *TBP* and *AK* were considered the optimal reference genes by BestKeeper. For temperature, the most stable genes were *RPS3* and *TBP* by geNorm and NormFinder, while the two optimal genes by BestKeeper analysis were *TBP* and *NADH*. Based on the three major statistic algorithms, *α-Tubulin* and *TBP* genes had a good performance under different conditions, while *HSP22* and *Actin* were the lowest expression stability genes according to the stability values. Our results indicate that the ranking of these reference genes were identical among treatments based on the analysis results of various software programs (geNorm, NormFinder and BestKeeper), these programs have different algorithms and different sensitivities toward co-regulated reference genes [19]. Therefore, each experimental investigation should establish which at least three reference genes are the most appropriate for the specific conditions [14]. According to the results of RefFinder, *α-Tubulin*, *TBP* and *AK* were the optimal reference genes.

*Tubulin* is one of several members of a small family of globular proteins, and is the major building block of microtubules in almost all eukaryotic cells, exhibited the most stable expression in the different conditions. Moreover, *Tubulin* had also been proved to be the normalized reference protein for Western blotting in *Drosophila* [25]–[27]. In this study, it was found to be the most stably expressed gene in the different treatment. *TBP* composed of transcription factor IID with *TBP*-associated factors, and is frequently used as a reference gene. Bansal *et al* recommend the *TBP* as a suitable HKG for efficient normalization among treatments, tissues, and developmental stages of *A. glycines* [28]. *AK* is the major phosphagen kinase in invertebrate groups, which has rarely been used as a reference gene in previous studies. In *Bombus terrestris*, *AK* was identified the most stable gene in the labial gland and fat body [8], and we found which the most stable gene was in different tissue (Figure S2). *RPL18* is located in the cytoplasm, which belongs to the L18E family of ribosomal proteins. Mamidala *et al* found that it is the most stable gene expression across all the tissue and developmental-stage in *Cimex lectularius* [8]. It is surprising that most of housekeeping genes performed poorly as reference genes in this study. Previously, *Actin* has been considered to be one of the best reference genes for assessing gene expression in many insects [29], [30], and *GAPDH* was also frequently used as a reference gene [11], [31]. However, they were not the best reference genes in the present analysis and several studies have demonstrated that the stability of *GAPDH* expression was low in certain conditions [32].These results further suggest that the expression stability of reference genes is affected by different experimental conditions.

This study was conducted to identify the stable reference gene(s) for qRT-PCR analyses of *D.suzukii* for studies of different biotic (developmental stage, tissue and population), and abiotic (photoperiod, temperature) conditions. After comprehensive consideration, we suggested that *α-Tubulin*, *TBP* and *AK* were the optimal reference genes. To our knowledge, this is the first systematic study to validate a set of different candidate reference genes for qRT-PCR in *D.suzukii*. The validation of reference genes in our study provides new information that will be useful for the accurate elucidation of the expression profiles of target genes. Furthermore, this study may also be useful for RNAi-based functional study and RT-PCR techniques that require reference gene for normalization in *D.suzukii.*

## Supporting Information

Figure S1 **Primer positions and ampliconic sequences are used for qRT-PCR.** The DNA sequences are shown from the 5′ to 3′ end, and the primer positions are underlined. The products were first amplified by underlined PCR and then sent to Invitrogen for sequencing.
(TIF)

Figure S2 **geNorm analysis of the expression stability of the 10 reference genes.** Average expression stability values (M) and ranking of the candidate reference genes as calculated by geNorm software. A lower average stability value indicates more stable expression.
(TIF)
