# Supplementary figures and images for: Identification and Validation of Reference Genes for Quantitative Real-Time PCR in Drosophila suzukii (Diptera: Drosophilidae)
Source: PLoS One. 2014 Sep 8;9(9):e106800. doi: 10.1371/journal.pone.0106800 (PMC4157791; doi:10.1371/journal.pone.0106800)

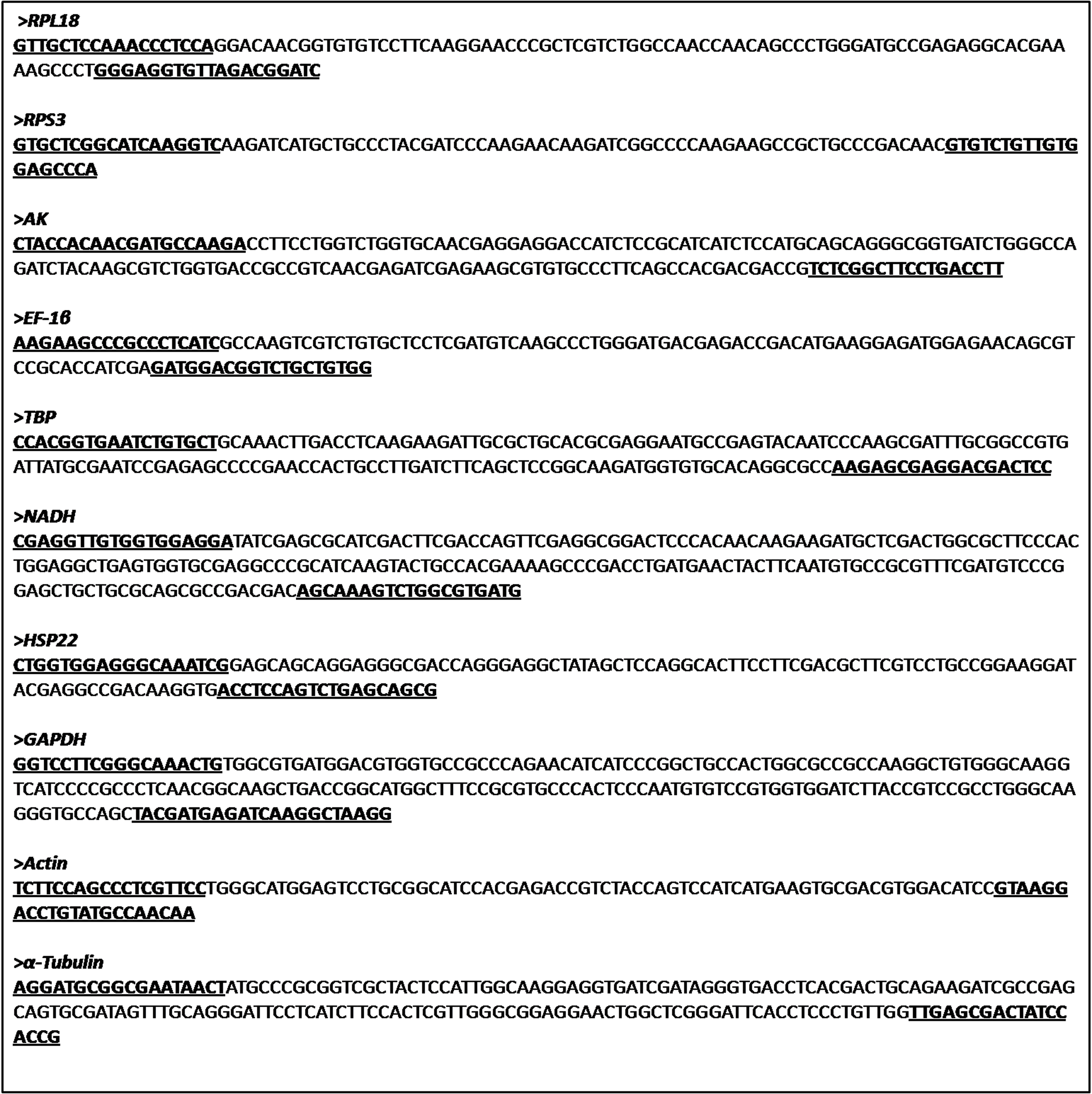

Supplement: Figure S1 — Primer positions and ampliconic sequences are used for qRT-PCR. The DNA sequences are shown from the 5′ to 3′ end, and the primer positions are underlined. The products were first amplified by underlined PCR and then sent to Invitrogen for sequencing. (TIF) [file pone.0106800.s001.tif]

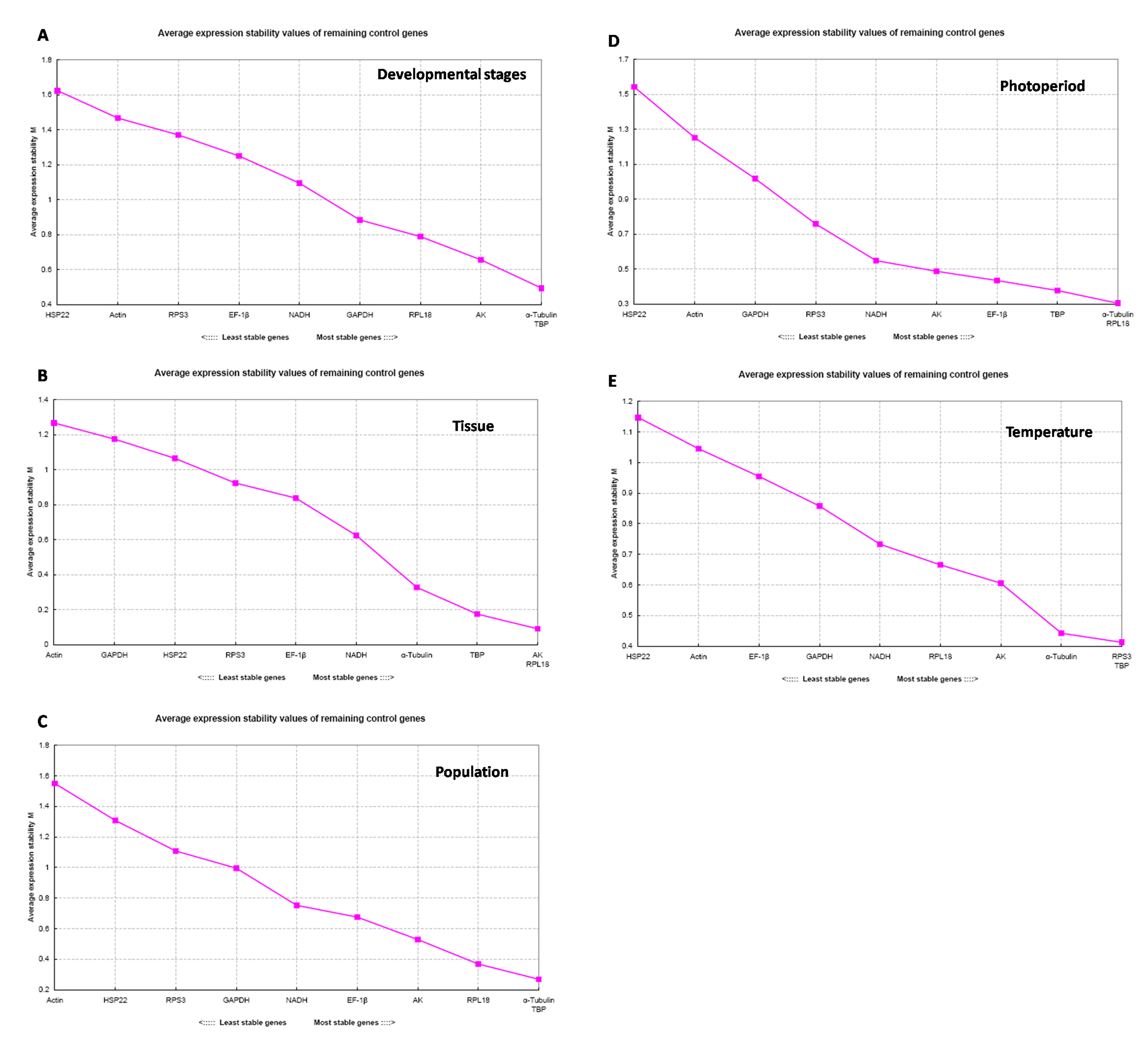

Supplement: Figure S2 — geNorm analysis of the expression stability of the 10 reference genes. Average expression stability values (M) and ranking of the candidate reference genes as calculated by geNorm software. A lower average stability value indicates more stable expression. (TIF) [file pone.0106800.s002.tif]
